# No Evidence for Acquired Mutations Associated with Cytochrome *bc*_1_ Inhibitor Resistance in 13,559 Clinical Mycobacterium tuberculosis Complex Isolates

**DOI:** 10.1128/AAC.01317-18

**Published:** 2018-12-21

**Authors:** Jan Rybniker, Thomas Andreas Kohl, Ivan Barilar, Stefan Niemann

**Affiliations:** a Department of Internal Medicine, University Hospital of Cologne, Cologne, Germany; b German Center for Infection Research (DZIF), Partner Site Bonn-Cologne, Cologne, Germany; c Center for Molecular Medicine Cologne, University of Cologne, Cologne, Germany; d Molecular and Experimental Mycobacteriology, Priority Area Infections, Research Center Borstel, Borstel, Germany; e German Center for Infection Research, TTU-TB, Borstel, Germany

**Keywords:** *Mycobacterium tuberculosis*, cytochrome *bc*_1_, lansoprazole, multidrug resistance, proton pump inhibitor

## LETTER

In 2017, tuberculosis (TB), caused by Mycobacterium tuberculosis, was responsible for an estimated 1.6 million deaths (1). The control of this pandemic is threatened because of a strong increase of multidrug-resistant M. tuberculosis (MDR-TB) (2). This emergence of difficult-to-treat strains requires the development of safe drugs with new mechanisms of action. A highly promising drug target is the cytochrome *bc*_1_ complex of the mycobacterial respiratory chain. Several chemically diverse cytochrome *bc*_1_ inhibitors with excellent antituberculous activity were identified in the past 5 years (3–9). One of these inhibitors is lansoprazole sulfide (LPZS), a close analogue and metabolite of the blockbuster drug lansoprazole (Prevacid), a gastric proton-pump inhibitor (PPI) (3). Mode-of-action studies revealed that lansoprazole targets cytochrome *bc*_1_ after prodrug conversion to LPZS in the host cell. Data on LPZS and its parent compound lansoprazole were recently exploited in a cohort study analyzing the incidence of TB among individuals taking lansoprazole for the treatment of gastric acid-related diseases (10). Lansoprazole is among the most widely sold drugs in the world, which enabled the evaluation of primary care patient records derived from the United Kingdom Practice Research Datalink (CPRD) (10). Intriguingly, this study demonstrated a statistically significant protective association between lansoprazole use and newly diagnosed TB disease. This is a surprising observation, since antimycobacterial activity of lansoprazole requires its conversion to LPZS. LPZS plasma concentrations during lansoprazole treatment are relatively low and may not exceed the *in vitro* MIC determined for M. tuberculosis (11). Nevertheless, the effect observed seems to be specific for lansoprazole and the antimycobacterial activity of lansoprazole analogues, since antacid treatment with two other PPIs (omeprazole and pantoprazole) was not protective against TB infection. Omeprazole and pantoprazole provided excellent controls in this study, since neither of the drugs possesses antimycobacterial activity due to structural restrictions (3).

These clinical data raise concerns on the susceptibility of clinical M. tuberculosis isolates to LPZS and other cytochrome *bc*_1_ inhibitors. Single-agent therapy and suboptimal dosing are well-known drivers of antibiotic resistance, as demonstrated for acquired fluoroquinolone resistance in M. tuberculosis isolates, which was associated with the receipt of fluoroquinolones prior to the TB diagnosis (12, 13). Widespread use of lansoprazole may have already selected for resistant strains, which would in turn hamper future clinical exploitation of these drugs in the fight against MDR-TB.

For this reason, we investigated cytochrome *bc*_1_ inhibitor resistance mutations in M. tuberculosis complex (MTC) whole-genome data derived from clinical isolates. Resistance in most cytochrome *bc*_1_ inhibitors identified so far is caused by single nucleotide polymorphisms (SNPs) of *qcrB* (Rv2196), causing mutations in the ubiquinol oxidation (QP) site of mycobacterial cytochrome *bc*_1_ (3–5, 9).

A total of 13,559 MTC next-generation sequencing data sets were analyzed with the MTBseq pipeline, i.e., reads were mapped to the H37Rv reference genome (GenBank identifier [ID], NC_000962.3) with the alignment program BWA, and mappings were refined and processed with the GATK and SAMtools toolkits (15). The samples were derived from both prospective and targeted collections of isolates and originated from countries around the world, with the vast majority of samples collected between 2010 and 2018. The collection contains strains from all lineages of the MTC (Table 1). For variant detection in mapped reads, we used the MTBseq default minimum thresholds of at least 4 reads coverage in both forward and reverse orientations, at least 4 reads calling the allele with at least a Phred score of 20, and 75% allele frequency (https://github.com/ngs-fzb/MTBseq_source). All data sets reached at least a mean coverage depth of 50-fold, with at least 95% of the reference genome covered with sufficient quality to meet variant detection thresholds.

**TABLE 1:** Phylogenetic lineages of *Mycobacterium tuberculosis* complex strains screened for cytochrome *bc*_1_ inhibitor resistance-associated mutations

MTC phylogenetic lineage^*a*^	No. of strains
East African, Indian (1)	424
East Asian, Beijing (2)	4,017
Delhi-CAS (3)	922
Euro-American (4)	7,537
West Africa, 1 (5)	183
West Africa, 2 (6)	192
Ethiopia (7)	7
Animal pathogens	265
M. canettii	12

a Numbers in parentheses indicate the lineage number (14).

Among all detected variants, we screened for any nonsynonymous SNP in codons 176, 182, 312, 313, 317, 342, and 396 of the *qcrB* gene (L176X, S182X, W312X, T313A, A317X, M342X, and A396X, respectively).

Among the 13,559 MTC genomes, there was only one Mycobacterium bovis strain which contained a T313A mutation causing resistance to Q203, a cytochrome *bc*_1_ inhibitor under investigation in clinical trials (9). For the remaining 13,558 MTC genomes, we detected wild-type sequences for the above-mentioned *qcrB* codons.

Our observations clearly show that extensive and worldwide use of proton pump inhibitors in the past decades did not lead to high prevalence of cytochrome *bc*_1_ resistance mutations in a representative number of clinical MTC isolates. This indicates that further clinical development of these promising antibiotics should not be compromised by prior lansoprazole treatment of people infected with M. tuberculosis.
